# Alleviating effects of gut micro-ecologically regulatory treatments on mice with constipation

**DOI:** 10.3389/fmicb.2022.956438

**Published:** 2022-08-09

**Authors:** Yueming Zhao, Qingjing Liu, Yanmei Hou, Yiqing Zhao

**Affiliations:** ^1^Key Laboratory of Dairy Science, Ministry of Education, Department of Food Science, Northeast Agricultural University, Harbin, China; ^2^Hyproca Nutrition Co., Ltd., Changsha, China; ^3^Hunan Provincial Key Laboratory of Food Science and Biotechnology, Changsha, China

**Keywords:** constipation, probiotics, postbiotics, synbiotics, gut microbial regulation, short-chain fatty acids

## Abstract

Treatments targeted for gut microbial regulation are newly developed strategies in constipation management. In this study, the alleviating effects of gut micro-ecologically regulatory treatments on constipation in mice were investigated. Male BALB/c mice were treated with loperamide to induce constipation, and then the corresponding intervention was administered in each group, respectively. The results showed that administration of mixed probiotics (MP), a 5-fold dose of postbiotics (P5), both synbiotics (S and S2), as well as mixed probiotics and postbiotics (MPP) blend for 8 days shortened the time to the first black stool, raised fecal water content, promoted intestinal motility, and increased serum motilin level in loperamide-treated mice. Furthermore, these treatments altered gut microbial composition and metabolism of short-chain fatty acids (SCFA). Based on linear regression analysis, SCFA was positively correlated with serum motilin except for isobutyrate. It suggested gut microbial metabolites affected secretion of motilin to increase gastrointestinal movement and transportation function and thus improved pathological symptoms of mice with constipation. In conclusion, the alteration of gut micro-ecology is closely associated with gastrointestinal function, and it is an effective way to improve constipation *via* probiotic, prebiotic, and postbiotic treatment.

## Introduction

Constipation, a worldwide digestive disorder, impacts around 14% of people in different regions (Black and Ford, 2018). Patients with constipation usually show symptoms like reduced frequency of defecation, hard or lumpy stools, a sensation of incomplete evacuation, and abdominal bloating and pain (Mearin et al., 2016). Chronic constipation not only hurts patients’ health but also increases the burden on the social health care system (Sharma and Rao, 2017). Conventionally, laxative drugs are widely used for the treatment of astriction, which can prompt defecation soon after administration, but the effects are less likely to exist when the drug use is ceased (Smith, 1973; Corazziari, 1999; Wang et al., 2020). Thus, laxative abuse probably leads to drug dependence and may deteriorate the symptom of constipation. Understanding the mechanisms of astriction has become the key aspect of developing new therapies.

Studies in recent years have shown the role of the gut microbiome in the pathogenesis of constipation. Zoppi et al. (1998) compared the composition of the fecal sample from children with constipation as well as their healthy counterparts and found that clostridia were more abundant in the constipated subjects (Zoppi et al., 1998). *Clostridium difficile* is the main cause to induce nosocomial diarrhea in children (Sadeghifard et al., 2010). Furthermore, in a children cohort with autism spectrum disorder (ASD), the proportion of *Clostridium* spp. including *Clostridium paraputri*, *Clostridium bolteae*, and *Clostridium perfringens* in the feces of was significantly higher than in neurotypical children, and especially *Clostridium diffiicile* and *Clostridium clostridiioforme* were not found in neurotypical children (Kandeel et al., 2020). This suggests that *Clostridium* spp. play an important role in affecting the development of diseases including neurological and intestinal disorders during childhood. Adult patients with functional constipation presented lower abundances of *Bacteroides*, *Roseburia*, and *Coprococcus* compared with healthy volunteers (Shin et al., 2019), and a pediatric study showed that the genus *Prevotella* decreased in constipated obese children compared with non-constipated obese children, while *Blautia*, *Coprococcus*, and *Ruminococcus* increased (Zhu et al., 2014). Animal experiments reported a reduced abundance of *Bacteroides*, *Lactobacillus*, *Desulfovibrio, and Methylobacterium*, and enriched *Clostridium* and *Akkermansia* (Cao et al., 2017; Wang et al., 2017a; Yi et al., 2019). Cao et al. transferred the feces of constipated patients to the intestine of germ-free mice and the recipient formed constipation-like symptoms, including reduced intestinal peristalsis and fecal water content (Cao et al., 2017). Although the character of the gut microbiome in constipated subjects differs across different researches, gut microbial dysbiosis probably contributes to the pathogenesis of constipation.

Intestinal health lays a great reliance on the gut microbiome (Sekirov et al., 2010). Gut microbiota affects the development of constipation *via* modulating the intestinal functions and communication with the central nervous system (Simon et al., 2021). Regulating gut microecology has been a newly developed strategy for constipation treatment. Probiotics, prebiotics, and synbiotics have been reported as dietary supplements to regulate immune responses, gut microbial composition, and metabolisms (Ştefãnuţ et al., 2015; Kamiński et al., 2020), and especially microbial metabolites contribute to the improvement of gastrointestinal functions and interaction between the gut and central system *via* cell signaling pathways (Mitrea et al., 2022). Probiotics supplement is one of the most effective measures to regulate gut microbiota. Many studies have concluded that probiotic consumption helps to change the gut microbial structure and alleviate constipation in humans (Dimidi et al., 2014; Martinez-Martinez et al., 2017; Huang et al., 2018) and animal models (Wang et al., 2017a,^2020^; Inatomi and Honma, 2021; Wang R. et al., 2021). Meanwhile, prebiotics, which can promote the growth of beneficial bacteria in the intestine, has been reported to improve constipation-associated disorders and partly change the gut microbial composition in adults (Yu et al., 2017; Chu et al., 2019). Present literature has demonstrated postbiotics consist of cell corpses, cell components, cell-wall fractions, cellular secretions, and metabolites, that are from probiotics and if received in sufficient quantity possess health effects on the host (Sabahi et al., 2022). In this research, the effects of mixed probiotics, postbiotics, synbiotics, and probiotics plus postbiotics on loperamide-induced constipation in BALB/c mice were compared, and their influence on the hormonal level, fecal short-chain fatty acids (SCFA), as well as gut microbial composition were sequenced to explore the potential targets of their constipation-alleviating function.

## Materials and methods

### Preparation of experimental formulation

The experimental formulation used in this study was a mixture of probiotics, prebiotics, postbiotics, and other nutritional components. Probiotics, prebiotics, and postbiotics were all purchased from Aunulife Biotechnology Co., Ltd. (Changsha, China). All these powdered products were resuspended or dissolved in saline before use. MP consisted of 6 strains (*Bifidobacterium animalis* subsp. *animalis* BB-115, *B. longum* subsp. *infantis* BLI-02, *Lacticaseibacillus rhamnosus* MP108, *Ligilactobacillus salivarius* AP-32, *L. rhamnosus* F-1, and *Lactiplantibacillus plantarum* LPL28) with a total colony-forming unit of 2.0 × 10^10^. The prebiotics contained lactitol, fructooligosaccharides, inulin, and stachyose, and postbiotics consisted of isolated soy protein and 4 strains including *L. plantarum* LPL28, *L. salivarius* AP-32, *B. longum* subsp. *infantis* BLI-02, and *Lactobacillus acidophilus* TYCA06. After fermentation, the supernatant of 4 strains was extracted *via* centrifuging, and then it was mixed with isolated soy protein after heat-kill sterilization. The mixture was made with postbiotics after heat-kill sterilization and spay-drying. Other components used as nutritional supplements were lactose, vitamin C, psyllium seed husk, and some fruit and vegetable powder.

### Animals and experimental design

Eight-week-old male BALB/c mice (*n* = 64) were purchased from Vital River Laboratory Animal Technology Co., Ltd. (Beijing, China). Mice were fed in a specific pathogen-free environment at a room temperature of 25°C ± 2°C and humidity of 50% ± 5% with a 12-h light–dark cycle, having free access to drinking water and standard chow. After 1 week of adaptation, mice were randomly divided into 8 groups (*n* = 8/group), containing the control group (control), model group (model), six constipated groups treated with mixed probiotics (MP), postbiotics (P and a 5-fold dose of P, P5), synbiotics (S and a 2-fold dose of S, S2), and probiotics plus postbiotics (MPP), and details of the experimental design were listed in Table 1. The Control group was orally treated only with saline per day during the experimental period by gavage, while the other groups were treated every day with loperamide (10 mg/kg body weight), and with corresponding products 1 h after loperamide administration. The treatments were carried out by gavage and lasted for 8 days. After the intervention period, animals were anesthetized with pentobarbital (50 mg/kg body weight) (Wang et al., 2010) and were then sacrificed by exsanguination. All protocols for the animal experiment were approved by the Ethics Committee of Jiangnan University [JN. No20201130b0720301(355)] and were in accord with the European Community guidelines (directive 2010/63/EU).

**TABLE 1:** Experimental design of the animal trail.

Groups	Treatment	Dose (mg/day)	Volume (μL/day)	Vehicle
Control	Saline	–	200	Saline
Model	Saline	–	200	Saline
MP	Mixed probiotics	–	200	Saline
P	Postbiotics	2.5	200	Saline
P5	Postbiotics	0.5	200	Saline
MPP	Mixed probiotics plus postbiotics	1.5	200	Saline
S	Synbiotics	12.3	200	Saline
S2	Synbiotics	24.6	200	Saline

### Defecation test

Defecation status was represented by first defecation time and fecal water content. Mice were treated with activated carbon meal 1 h after treatments on the eighth day, and then the time to the first black stool of each mouse was measured according to Wang et al. (Cao et al., 2017). Feces were then collected in individual sterile EP tubes on ice and the water content was determined as described by Lee et al. (2012).

### Determination of intestinal propulsion rate

Gastrointestinal (GI) motility was determined as previously described with a modification (Verdu et al., 2009). Mice were fasted from 6:00 p.m. for 18 h on the eighth day and were then treated with loperamide (treated groups) or saline (control group), and activated carbo meal, after which mice were anesthetized with pentobarbital (50 mg/kg body weight) and executed by exsanguination (Wang et al., 2010). The blood sample was collected immediately centrifuged at 3000 × *g*, 4°C for 15 min to obtain serum. The entire intestine was carefully removed out of the abdomen and put on blotting paper. The distance from the pylorus to the end of the darkened intestine as well as the entire length of the intestine was measured. The intestinal propulsion rate was calculated by the ratio of the length of the black intestine to the entire intestinal length.

### Serum cytokine detection

Serum samples were divided into 2 to 3 individual sterile EP tubes and then stored at −80°C. Cytokines containing IFN-α, TGF-β1, TNF-α, IL-1β, IL-6, IL-10, and IL-17A were determined using a MILLIPLEX MAP Kit Mouse Cytokine/Chemokine Magnetic Bead Panel (EMD Millipore Corporation, Billerica, MA, United States) according to the manufacturer’s instruction and performed with a Luminex MAGPIX System (Luminex, Austin, TX, United States).

### Quantification of fecal short-chain fatty acids

Fecal samples (∼50 mg) were soaked with saturated NaCl solution and acidified with sulfuric acid (10%). Total SCFAs extracted with diethyl ether. Determination of the concentration of SCFA was carried out through a gas chromatography-mass spectrometry system (GCMS-QP2010 Ultra system, Shimadzu Corporation, Japan) according to Wang et al. (2017a). The concentration of total SCFA was calculated as the sum of acetic, propionic, butyric, isobutyric, valeric, and isovaleric acids.

### Analysis of fecal microbiome

Total DNA was extracted from fecal samples with a FastDNA Spin Kit for Soil (MP Biomedicals, catalog no. 6560-200) following the manufacturer’s instructions. The V4 region of the 16S rRNA gene was amplified using a specific primer (forward primer, 5′-AYT GGG YDT AAA GNG-3′; reverse primer, 5′-TAC NVG GGT ATC TAA TCC-3′) by polymerase chain reaction (PCR) as previously described. Then the PCR products were purified, quantified, and sequenced as previously described by Zhao et al. (2018). Afterward, the 16S rRNA sequence data were analyzed using the QIIME2 pipeline as previously described (Zhao et al., 2018).

### Statistical analysis

Results were all expressed as mean ± SD. One-way ANOVA and Dunnett’s multiple comparison test were applied for the comparison between groups. Comparisons and linear regression analysis were carried out using GraphPad Prism version 8.0.2 (GraphPad Software Inc., United States). Values with *p* < 0.05 were considered significantly different. Pearson’s correlation analysis was conducted in MetaboAnalyst 5.0.^1^

## Results

### The synbiotics improved defecation status and gastrointestinal transit

**FIGURE 1:**
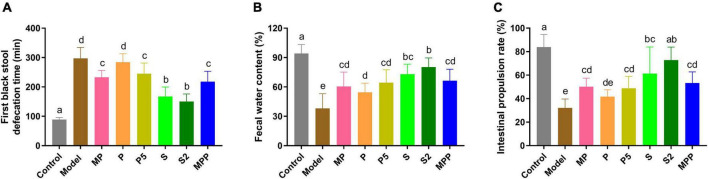
The differences in **(A)** time to the first black stool, **(B)** fecal water content, and **(C)** intestinal propulsion rate among groups. Values are expressed as mean ± SEM (*n* = 8). Results were compared by one-way ANOVA followed Duncan’s multiple comparison test. Different letters represent significant differences (*p* < 0.05).

As shown in Figure 1A, compared with the control group, a 2.3-fold increase in the time to the first black stool was observed in the model group (*p* < 0.0001), suggesting the formation of constipation due to loperamide treatment. The figure for S and S2 treatments decreased to approximately 50% of that of the model group. MP, P5, and MPP treatments partly shortened the defecation time, while P treatment showed little effect on it. Fecal water content in the model group decreased in comparison with the control group, which was nearly restored in S and S2 groups (Figure 1B). MP, P5, and MPP partly raised fecal water content, while the effect of P treatment was less significant than other treatments (Figure 1B). As shown in Figure 1C, the intestinal propulsion rate in the model group was significantly lower than that in the control group. Administration of S2 restored the decrease in intestinal propulsion rate, while the treatment with S partly inverted the decrease. MP, P5, and MPP slightly promoted the intestinal propulsion rate. In contrast, P treatment did not affect the intestinal propulsion rate in constipated mice.

### The synbiotics changed serum hormones relevant to gastrointestinal motility

**FIGURE 2:**
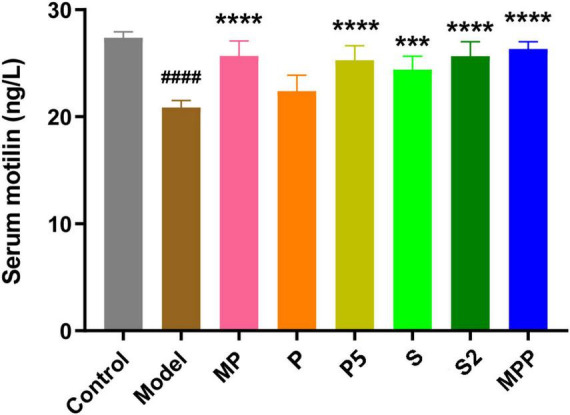
The differences in serum motilin level among groups. Values are expressed as mean ± SEM (*n* = 8). Results were compared by one-way ANOVA and Dunnett’s multiple comparison test: ***p < 0.001, ****p < 0.0001 compared with the model group; **^####^***p* < 0.0001 compared with the control group.

Motilin, a peptide hormone released from enterochromaffin cells, promotes GI contraction and digestive motility (Kamerling et al., 2003). A lack of motilin is associated with both normal transit constipation and slow transit constipation (Wang Y. Y. et al., 2021). As shown in Figure 2, compared with the control group, a significant decrease in serum motilin level was recorded in the model group (*p* < 0.0001). All the interventions except for P restored the serum motilin level in constipated mice. Furthermore, all the interventions did not change the level of proinflammatory cytokines in serum, including IFN-α, TGF-β1, TNF-α, IL-1β, IL-6, IL-10, and IL-17A (Table 2).

**TABLE 2:** Serum cytokine concentration in different groups (ng/L).

Groups	IL-6	IL-10	IL-17A	TNF-α	IFN-α	TGF-β 1	IL-1β
Control	15.21 ± 2.34	70.82 ± 7.02	1.00 ± 0.16	70.87 ± 10.99	3.42 ± 0.46	22.44 ± 2.21	10.23 ± 0.84
Model	13.80 ± 0.92	69.10 ± 10.50	1.17 ± 0.20	67.06 ± 4.04	4.04 ± 0.41	23.36 ± 4.10	11.49 ± 1.94
MP	13.85 ± 1.49	70.50 ± 8.84	1.11 ± 0.17	71.52 ± 7.74	3.82 ± 0.64	24.94 ± 5.01	11.28 ± 1.66
P	13.58 ± 1.34	70.39 ± 9.56	1.10 ± 0.18	70.91 ± 8.33	3.82 ± 0.58	22.64 ± 1.21	10.57 ± 0.98
P5	13.50 ± 1.96	70.31 ± 7.72	1.18 ± 0.13	67.04 ± 8.53	4.28 ± 0.49	21.94 ± 2.85	10.28 ± 1.78
MPP	14.64 ± 1.66	67.94 ± 8.65	1.20 ± 0.25	65.25 ± 7.01	4.12 ± 0.32	22.93 ± 3.83	10.65 ± 0.90
S	13.87 ± 2.32	70.34 ± 6.62	1.16 ± 0.14	71.34 ± 5.69	4.14 ± 0.17	22.78 ± 2.58	10.29 ± 1.16
S2	13.26 ± 1.64	71.80 ± 7.24	1.18 ± 0.24	68.56 ± 6.66	4.17 ± 0.23	24.50 ± 2.49	10.99 ± 1.55

Values were expressed as mean ± SD. Results were compared by one-way ANOVA and Dunnett’s multiple comparison test.

### The synbiotics changed gut microbial composition in mice

**FIGURE 3:**
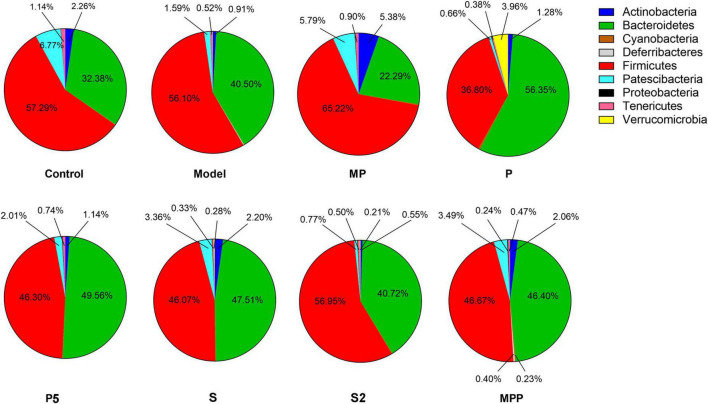
The differences in the relevant abundance of gut microbial phyla among groups.

16S rRNA sequencing results showed that Firmicutes and Bacteroidetes were the two dominating phyla in fecal samples. As shown in Figure 3, after loperamide treatment, the relative abundance of Actinobacteria decreased from 2.26 to 0.91%, and the figure for Patescibacteria dropped from 6.77 to 1.59%, while Bacteroidetes increased from 32.38 to 40.50%. MP administration upregulated Actinobacteria, Patescibacteria, and Firmicutes in constipated mice, and down-regulated Bacteroidetes. The proportion of Verrucomicrobia and Bacteroidetes increased in the P group compared with the model group, while Firmicutes decreased. A significant increase in Actinobacteria was observed in S and MPP groups.

**FIGURE 4:**
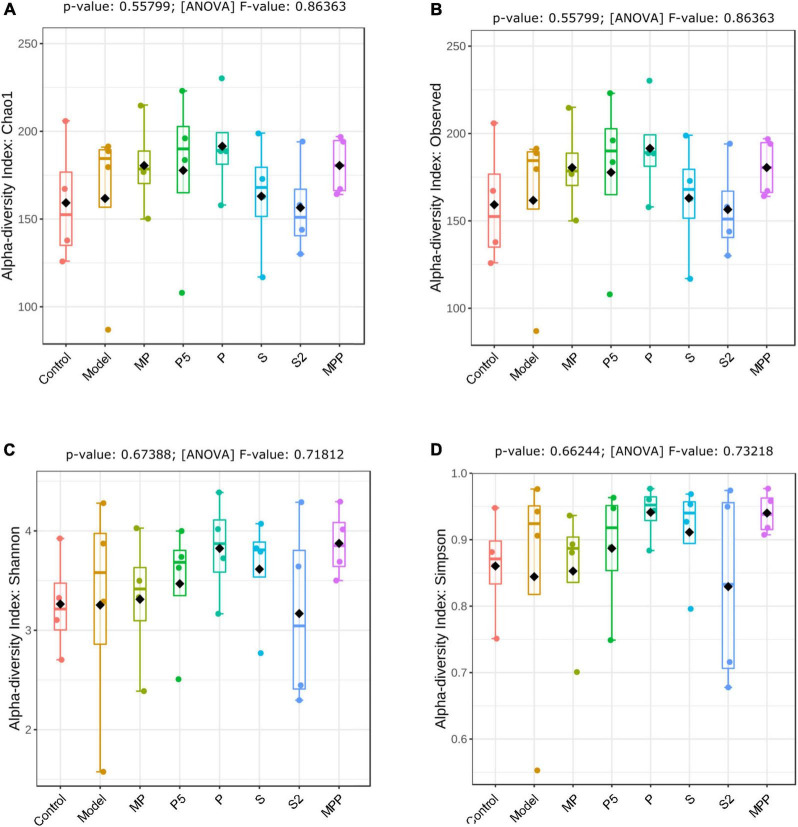
The differences in **(A)** Chao1 index, **(B)** observed OTUs, **(C)** Shannon index, and **(D)** Simpson index of fecal microbiome among groups. Results were compared by one-way ANOVA and Dunnett’s multiple comparison test.

No significant change was observed between the control and model groups from the alpha-diversity index, including Chao1, Simpson, Shannon, as well as observed OTUs (Figures 4A–D). Treatments other than the synbiotics increased the Chao1 index and observed OTUs in mice (Figures 4A,B). Mice in the treated groups except for MP and S2 groups exhibited an obvious increase in Shannon and Simpson indexes (Figures 4C,D).

**FIGURE 5:**
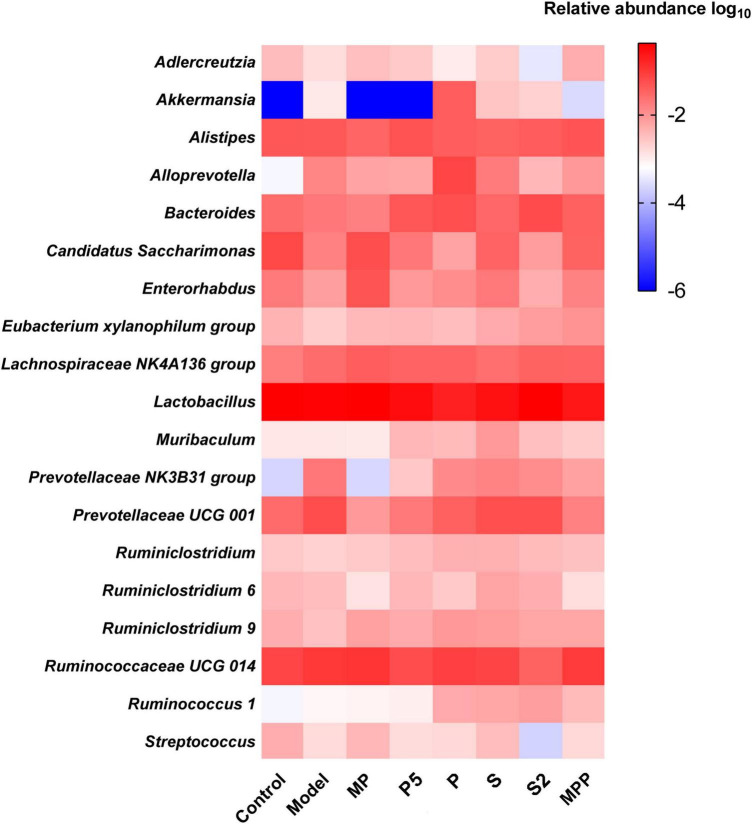
Heatmap of the top 20 fecal microbial genera among groups.

At the genus level, no apparent difference was found between the model and control groups except the increased Akkermansia in the model group (Figure 5). Ruminiclostridium 6, Ruminiclostridium 9, and Eubacterium xylanophilum group were more abundant in MP and MPP groups, in comparison with the model group, while the abundance of Alistipes was much lower (Figure 5). There was a rise in Ruminiclostridium 6, Ruminiclostridium 9, Ruminococcus 1, Ruminiclostridium, and Bacteroides, as well as a decrease in Alistipes in the P, P5, S, and S2 groups compared with the model group (Figure 5).

### The synbiotics changed fecal short-chain fatty acids concentration in mice

The concentration of fecal SCFA is an important implication of intestinal motility. As shown in Table 3, loperamide administration decreased the level of fecal acetate in mice, and this decrease was reversed in the MP, P5, S, S2, and MPP groups. While the decline in fecal propionate was restored in all these groups. Butyrate in stool was less in the model group than in the control group, which was reversed in the MP, P5, S, and S2 groups. A significant decrease in fecal valerate could be found in the model group, and this reduction was reversed by MP, S2, and MPP treatments. No obvious difference in fecal isobutyrate and isovalerate was observed between the control and model groups. Meanwhile, the level of isovalerate in stool increased in the MP, S, and S2 groups.

**TABLE 3:** Fecal SCFA concentration in different groups (pmol/mg).

	Acetate	Propionate	Butyrate	Isobutyrate	Valerate	Isovalerate
Control	89.568 ± 22.712	15.094 ± 1.767	15.958 ± 3.020	3.354 ± 0.772	2.446 ± 0.228	2.262 ± 0.347
Model	54.733 ± 15.559^#^	8.133 ± 1.368^#^	6.271 ± 3.423^#^	2.488 ± 0.464	1.511 ± 0.169^#^	1.632 ± 0.381
MP	103.986 ± 18.477**	13.912 ± 2.841*	15.066 ± 4.123*	3.487 ± 0.806	3.140 ± 0.492***	3.042 ± 0.974**
P	54.706 ± 11.628	8.449 ± 1.303	7.616 ± 3.335	2.278 ± 0.493	1.506 ± 0.429	1.511 ± 0.226
P5	76.496 ± 11.071*	16.669 ± 3.743*	17.247 ± 6.501*	2.597 ± 0.334	2.322 ± 0.418	1.764 ± 0.485
MPP	106.951 ± 19.109**	19.486 ± 6.669**	19.129 ± 7.339*	3.608 ± 0.569	2.354 ± 0.847	2.803 ± 0.501*
S	116.413 ± 16.732***	22.318 ± 10.578**	20.168 ± 6.779**	3.664 ± 0.616	2.695 ± 0.427**	3.193 ± 0.631*
S2	100.315 ± 15.478**	17.006 ± 3.752*	16.817 ± 8.198	3.262 ± 0.948	2.703 ± 0.607**	2.407 ± 0.323

Values are expressed as mean ± SD. Results were compared by one-way ANOVA and Dunnett’s multiple comparison test: *p < 0.05, **p < 0.01, ***p < 0.001 compared with the model group; ^#^p < 0.05 compared with the control group.

**FIGURE 6:**
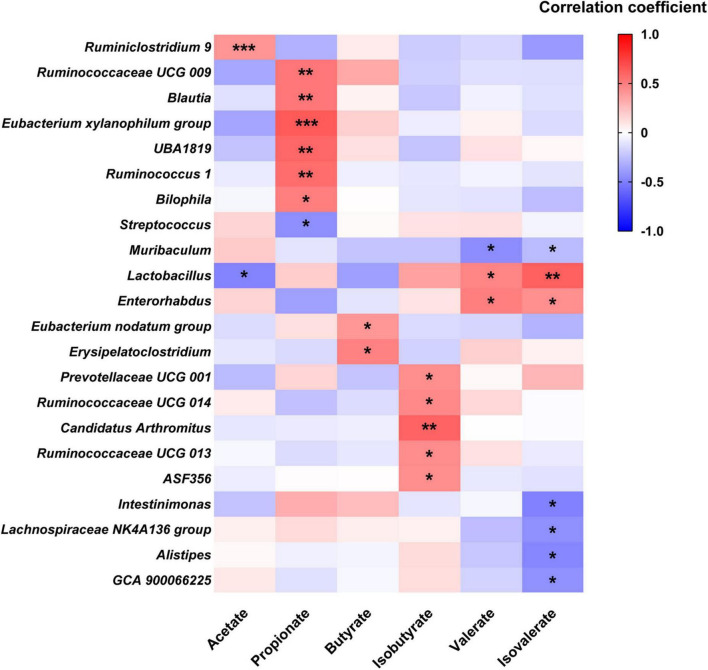
Heatmap of Pearson’s correlation analysis between SCFA and fecal microbial genera. *p < 0.05, ** *p* < 0.01, *** *p* < 0.001 statistical significance of Pearson’s correlation analysis.

To explore the relationship between fecal SCFA and gut microbial genera, Pearson’s correlation analysis was carried out. As shown in Figure 6, fecal acetate was positively correlated with Ruminiclostridium 9 and negatively associated with Lactobacillus. Fecal propionate level was positively related to six genera, including Ruminococcus 1 and Blautia, while negatively correlated with Streptococcus. Butyrate was just positively associated with Eubacterium nodatum group and Erysipelatoclostridium, while isobutyrate was positively correlated with five genera like Prevotellaceae UCG 001, Ruminococcaceae UCG 013, and Ruminococcaceae UCG 014. In contrast, valerate was positively related to Lactobacillus and Enterorhabdus, while negatively associated with Muribaculum. A positive correlation was observed between isovalerate and two genera: Lactobacillus and Enterorhabdus, while a negative one was recorded between isovalerate and five genera, including Muribaculum, Intestinimonas, Lachnospiraceae NK4A136 group, Alistipes, and GCA 900066225, respectively.

**FIGURE 7:**
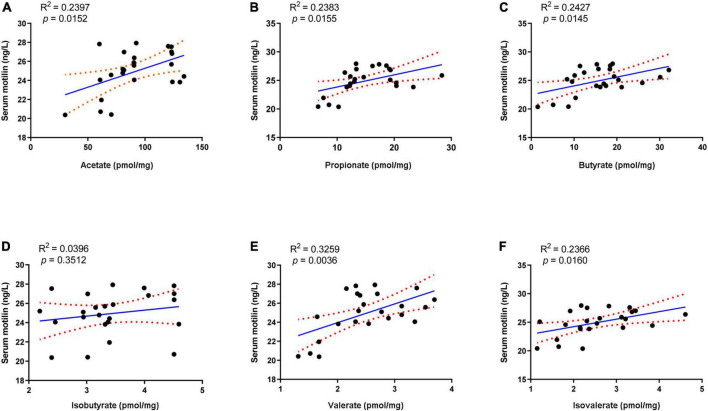
Linear regression analysis between serum motilin level and **(A)** acetate, **(B)** propionate, **(C)** butyrate, **(D)** isobutyrate, **(E)** valerate, and **(F)** isovalerate.

Linear regression analysis was applied to analyze the correlation between serum motilin and fecal SCFA. Among these SCFA, acetate, propionate, butyrate, valerate, and isovalerate exhibited positive correlations with serum motilin levels, while isobutyrate was not statistically correlated with that (Figure 7).

## Discussion

Constipation is a functional GI disorder that is conventionally recognized by a decrease in defecation frequency, fecal water content, as well as GI motility. In recent years, the strategies based on gut microecological regulation, such as the supplementation of probiotics, prebiotics, and synbiotics, have drawn more attention. In this study, the effects of probiotics, postbiotics, and synbiotics were investigated in a mice model of spastic constipation, which was induced by loperamide administration (Salminen et al., 1998).

Loperamide, an agonist of μ-opioid receptors, prevents the release of acetylcholine and prostaglandin, thus inhibiting intestinal peristalsis and leading to prolonged retention of intestinal contents (Wang et al., 2017b). Our results showed that loperamide treatment caused typical symptoms of constipation, including an apparent increase in the time to the first black stool defecation and an obvious decrease in fecal water content. Administration of MP, P5, S, S2, and MPP treatments shortened the time to the first black stool and increased fecal water content in loperamide-treated mice, suggesting alleviations of these interventions. Constipation in mice was further confirmed by the decrease in intestinal propulsion rate. These treatments promoted intestinal propulsion rate in constipated mice, indicating an improvement in GI motility. In contrast, P treatment slightly improved fecal water content and GI motility but failed to shorten the time to the first black stool. Therefore, P treatment was less potent in relieving constipation than P5, and this might be due to the differences in dose.

Motilin is secreted by endocrine cells in the duodenum, jejunum, and antrum (Polak et al., 1975), and plays an important role in maintaining GI motility by stimulating its receptors (Kamerling et al., 2003). In this study, a significant decrease in serum motilin was recorded in constipated mice in contrast to healthy mice. This helped to explain the reduction in GI motility in this mice model. All interventions except for P restored serum motilin levels in constipated mice. Thus, MP, P5, S, S2, and MPP treatments might contribute to the stability of intestinal motility by sustaining motilin levels.

Alterations in the fecal microbiome were detected in constipated control mice, including the decreased Actinobacteria and Patescibacteria as well as the raised Bacteroidetes at the phylum level, suggesting gut microbial alterations due to loperamide treatment. Treatment with MP upregulated Firmicutes and downregulated Bacteroidetes in constipated mice. It was previously reported that increased Bacteroidetes and decreased Firmicutes were associated with constipation in rodents (Wang et al., 2017b). Thus, the probiotics applied in this study might help to reduce the risk of constipation by regulating the ratio of Firmicutes to Bacteroidetes. As for diversity, treatments increased different alpha-diversity indicators, suggesting that these interventions contribute to stabilizing the gut microbiome in the host.

Apart from regulating gut microbiota, probiotics, prebiotics, and synbiotics can influence the fermentation products in the gut, such as SCFA (Wang et al., 2017b; Lan et al., 2020). SCFA is mainly formed by the bacterial fermentation of indigestible carbohydrates in the large intestine and can stimulate intestine contraction, thereby promoting GI motility (Niwa et al., 2002). Previous studies revealed an association between improved constipation and fecal SCFA concentration, especially those using microbial regulating strategies like supplementation of probiotics (Wang et al., 2017b), prebiotics (Lan et al., 2020), and dietary fiber (Niwa et al., 2002; Zhuang et al., 2019). Our results showed that fecal acetate, propionate, butyrate, and valerate decreased after loperamide treatment, and these decreases were inverted by the administration of MP (Table 3). MPP treatment restored the changes in acetate, propionate, and valerate during constipation. Both S and S2 treatments restored the reduction in acetate, propionate, and butyrate, and increased isovalerate in the feces, while S2 upregulated fecal valerate in constipated mice. In contrast, P5 treatment raised acetate, propionate, and butyrate in stool, while P treatment failed to change fecal SCFA profile in constipated mice. Meanwhile, the correlation analysis showed that the SCFA other than isobutyrate was positively correlated with serum motilin, indicating that increased intestinal SCFA may contribute to upregulated motilin synthesis. Therefore, the alterations in SCFA in the gut after the interventions except for P might contribute to promoting GI motility and alleviating constipation.

Alterations in gut microbial genera probably lead to changes in fecal SCFA profile. In this study, the relative abundances of *Ruminiclostridium 9* and *Eubacterium xylanophilum group* were increased after MPP administration, respectively, while *Alistipes* was reduced. Pearson’s correlation analysis suggested that fecal acetate concentration was positively associated with *Ruminiclostridium* 9, and propionate was positively associated with the *Eubacterium xylanophilum group*, while *Alistipes* negatively related to isovalerate. *Ruminiclostridium 9* and the *Eubacterium xylanophilum group* were previously reported to be SCFA producers in the gut (Guo et al., 2018). Hence, the rise in these two genera in the gut might be favorable for improving SCFA production, thus promoting intestinal movement after MPP intervention. Treatments with P, P5, S, and S2 upregulated genera *Ruminiclostridium 9*, *Ruminiclostridium*, *Ruminococcus 1*, and *Bacteroides* in feces. *Ruminococcus 1*, which was identified as an SCFA producer (Louis and Flint, 2017), positively correlated with propionate in our results. According to Jacobson et al., upregulation of *Bacteroides* may help to raise propionate production, although fecal propionate did not show a significant correlation in this study (Jacobson et al., 2018). Accordingly, increases in *Ruminiclostridium 9*, *Ruminiclostridium*, *Ruminococcus 1*, and *Bacteroides* induced by P, P5, S, and S2 administrations might contribute to stimulating GI motility and ameliorating constipation.

## Conclusion

The present study showed that the MP, P5, S, S2, and MPP treatments shortened the time to the first black stool, increased fecal water content and GI motility, and raised serum motilin levels in mice. Meanwhile, these interventions changed the gut microbial structure and increased fecal SCFA concentration, which contributed to their alleviating effects on constipation.

## Data availability statement

The original contributions presented in this study are publicly available. This data can be found here: https://www.ncbi.nlm.nih.gov/bioproject/PRJNA842449; accession number: PRJNA842449.

## Ethics statement

The animal study was reviewed and approved by the Ethics Committee of Jiangnan University [JN. No. 20201130b0720301 (355)].

## Author contributions

YQZ conceived the project. YMZ completed the experiments and wrote the manuscript. QL and YH edited the manuscript. All authors contributed to the article and approved the submitted version.

## Footnotes

1
https://www.metaboanalyst.ca/
